# Nanocrystalline Cellulose Reinforcement and Constrained Expansion to Enhance Mechanical Performance of Rigid Polyurethane Foams for Sandwich Panel Applications

**DOI:** 10.3390/ma18091950

**Published:** 2025-04-25

**Authors:** Marcelo Jorge Bach, Kelvin Techera Barbosa, Cristiane da Silva Fonseca, Darci Alberto Gatto, Rafael Beltrame, André Luiz Missio, Jalel Labidi, Rafael de Avila Delucis

**Affiliations:** 1 Post-Graduate Program in Environmental Sciences (PPGCAmb), Federal University of Pelotas, Pelotas 96010-610, Brazil; marcelojbach84@gmail.com (M.J.B.); darcigatto@yahoo.com (D.A.G.); beltrame.rafael@yahoo.com.br (R.B.); andreluizmissio@gmail.com (A.L.M.); 2 Postgraduate Program in Mining, Metallurgical and Materials Engineering, Federal University of Rio Grande do Sul, Porto Alegre 91501-970, Brazil; kelvintecherabarbosa@gmail.com (K.T.B.); cristianesilvafonseca@outlook.com (C.d.S.F.); 3 Postgraduate Program in Materials Science and Engineering (PPGCEM), Technology Development Center, Federal University of Pelotas (UFPel), Pelotas 96010-610, Brazil; 4 Chemical and Environmental Engineering Department, University of the Basque Country (UPV/EHU), 20018 San Sebastian, Spain

**Keywords:** nanocrystalline cellulose, dynamic mechanical analysis, foam confinement, bending strength, Oriented Strand Board, load-bearing capacity

## Abstract

This study aimed to assess the mechanical and morphological properties of rigid polyurethane foams (RPUFs) reinforced with cellulose nanocrystals (CNC) at varying concentrations, exploring also effects of expansion under confinement for use in sandwich panels. RPUFs with 1%, 3%, and 5% CNC were tested, with the 3% CNC content delivering the best combination of mechanical performance and cellular structure. While the RPUF with 5% CNC showed a 78% increase in cell length, its compressive strength dropped by 55%, likely due to CNC agglomeration. Confining the RPUF during expansion improved the density by 23%, which in turn led to an approximately 90% increase in core shear stress. Flexural tests revealed that confined panels exhibited better force-displacement responses, with core shear strength rising by 55% compared to unconfined panels. These results suggest that CNC-reinforced and confined RPUFs are well-suited for structural applications requiring both strength and insulation.

## 1. Introduction

Some rigid polyurethane foams (RPUFs) are widely recognized for their excellent properties, such as good thermal insulation, mechanical strength, and lightweight. These characteristics make them highly used in several industries, like aerospace, construction, and automotive [1,2,3]. As technology advances to increase their mechanical properties, RPUFs have become an attractive choice for sandwich panels, where two outer layers, or face sheets, are bonded to a lightweight core, typically the foam [4,5]. These panels are used in many engineering applications due to their high strength-to-weight ratios [1,3]. In general, RPUFs with improved mechanical performance could be used as the core material because of their low density and good insulating properties. However, in cases where the panel is subjected to higher mechanical stresses, traditional RPUFs may not be strong enough [2].

The need to enhance the mechanical properties of these materials has led researchers to explore the use of reinforcing agents. One of the most promising materials for this purpose is cellulose nanocrystals (CNCs) or nanocrystalline cellulose (CNC), which are derived from renewable resources, particularly wood, making them an environmentally friendly option. By using CNCs as a reinforcing agent, it is possible to reduce the reliance on petrochemical-based materials in the RPUFs [2,6]. This contributes to the sustainability of the material, aligning with global trends towards more eco-friendly construction materials. Life-cycle assessments have shown that using nanoparticles based on cellulose can reduce the carbon footprint of RPUF’s production by up to 50% [7]. This makes CNC-reinforced foams not only a high-performing material but also a greener alternative for future building materials.

The reinforcement of rigid polyurethane foams (RPUFs) with cellulose nanocrystals (CNCs) also represents a significant advancement in material science, offering notable improvements in both performance and sustainability. CNCs possess exceptional mechanical properties, such as high tensile strength and stiffness, which contribute to enhancing the structural integrity of the foam matrix [2,6]. When incorporated into RPUFs, CNCs act as reinforcing agents, leading to substantial improvements in properties like compressive strength, flexural stiffness, and overall dimensional stability. These enhancements not only increase the foam’s load-bearing capacity but also improve its resistance to deformation under stress, making CNC-reinforced RPUFs particularly suitable for demanding, high-performance applications such as automotive, aerospace, and construction industries. Additionally, CNCs contribute to better thermal insulation by promoting a more uniform cell structure, which helps to reduce heat transfer through the material. Beyond their mechanical and thermal benefits, CNCs are derived from renewable biomass, giving them a lower environmental footprint compared to traditional synthetic additives. This makes CNC-reinforced RPUFs an attractive option for eco-friendly and sustainable construction solutions, where performance, durability, and environmental responsibility are equally prioritized.

For instance, recent studies have demonstrated that CNCs can enhance the performance of RPUFs, including increases in compressive strength by up to 45% and over 30% in modulus [8,9,10]. Additionally, dynamic mechanical analyses (DMA) have shown that the storage modulus of CNC-reinforced foams can increase by up to 50%, providing better stiffness and load-bearing capacity [11]. Similarly, research by Zhou et al. [8] demonstrated an increase in compressive strength from 54 kPa to 117 kPa when CNC was added to bio-RPUFs with further enhancements in foam rigidity under cyclic loading. These mechanical benefits make CNC-reinforced foams particularly useful in industries where weight reduction without compromising strength is essential [4]. This means that panels with cores made with CNC-reinforced RPUFs can bear more load before deforming, which is particularly important in structural applications like flooring, roofing, and wall systems [5]. These characteristics also make CNC-reinforced RPUFs a promising option for long-term, high-performance applications [3,12].

CNCs not only enhance the mechanical performance of rigid polyurethane foams (RPUFs), but they also play a critical role in improving their thermal properties. By altering the cellular structure of the foam, CNCs contribute to a more efficient barrier against heat transfer. Recent studies have demonstrated that CNC-reinforced RPUFs exhibit significantly lower thermal conductivity compared to their unreinforced counterparts, making them highly effective for insulation applications. For example, in a study by Septevani et al. [13], the incorporation of just 0.4 wt.% CNCs resulted in a 5% reduction in thermal conductivity. This reduction represents a meaningful advancement in the performance of rigid foams, as it improves their ability to insulate against heat, ultimately enhancing energy efficiency. Such improvements are particularly valuable in building construction, where reducing energy consumption and improving insulation are key priorities for achieving sustainable and energy-efficient designs [14].

These gains in mechanical and thermal performance can be attributed to the influence of CNCs (cellulose nanocrystals) in the cellular structure of the foam, where they act as nucleating agents, leading to smaller and more uniformly distributed cells throughout the material [4]. This enhanced cell structure directly contributes to improved material properties by increasing the foam’s density and uniformity, which minimizes defects and weak points within the matrix. For example, scanning electron microscopy (SEM) images have revealed a reduction in average cell size from approximately 518 μm to 510 μm with the incorporation of just 0.4% CNCs by weight [10]. This subtle decrease in cell size results in a denser, more compact structure that not only enhances the mechanical strength of the rigid polyurethane foams (RPUFs) but also boosts their thermal insulation properties by reducing heat transfer through the material. Additionally, the more uniform cell distribution has been shown to improve the foam’s acoustic performance, as smaller, evenly dispersed cells create more effective barriers to sound transmission, making the material a strong candidate for applications requiring both mechanical resilience and enhanced thermal and acoustic insulation.

However, the improvements in mechanical and thermal properties are highly dependent on the proper dispersion and incorporation methods used, as well as the interaction between the CNCs and the polymer matrix [10,14]. Proper dispersion ensures that CNCs don’t agglomerate, which can weaken the structure, while good compatibility promotes stronger reinforcement. Optimizing CNC content in RPUFs is critical, as it directly affects both strength and thermal insulation. However, the ideal CNC loading is still not well-defined, with various factors like dispersion quality, compatibility, and loading levels influencing the final properties of the foam. Despite progress, further refinement is needed to determine the optimal balance.

One critical challenge is avoiding CNC re-agglomeration, which can disrupt the foam’s cellular structure and negatively affect mechanical properties. Ugarte et al. [15] stated that achieving a stable and effective CNC dispersion plays a key role in reinforcing polyurethane matrices, especially when renewable polyols are used. Furthermore, studies using ultrasonication, such as those by Septevani et al. [14], has found that optimizing the CNC dispersion method is essential to achieving uniform cell structures and enhanced nucleation. Thus, while the reinforcement of RPUFs with CNC shows great potential, the exact amount of CNC needed for optimal mechanical and thermal performance remains a topic of ongoing research. Various studies have suggested that the balance between CNC content and foam matrix integrity is delicate, with excess CNC leading to diminished returns due to aggregation or poor interaction with the foam matrix.

In addition to using reinforcing agents like CNCs, the process of confinement has gained attention as a method to further enhance the mechanical performance of RPUFs, ensuring certain applications. Confinement refers to the restricted expansion of the foam during its formation process, which leads to increased density and improved structural performance [16,17]. The use of confinement has proven to be a promising approach for addressing the limitations of RPUFs in high-stress applications. In sandwich panel construction, for example, confinement of the foam core can significantly enhance the panel’s load-bearing capacity and durability [17]. For instance, according to Kerche et al. [16], RPUFs confined to 50% of their free expansion volume displayed up to a 45% increase in compressive strength. This improvement can be attributed to the more compact and uniform cell distribution, which allows the foam to better resist mechanical deformation under load [17], particularly in demanding structural environments such as flooring systems and load-bearing walls [18]. The objective of this study is to explore the mechanical properties of CNC-reinforced RPUFs at different CNC weight fractions and use expansion under confinement to evaluate their feasibility for use in sandwich panels. 

## 2. Materials and Methods

### 2.1. Materials and Foam Manufacturing

The CNC used in this study was supplied by CelluForce Industry, Montreal, QC, Canada, in the form of a spray-dried powder. The product is characterized by a white to off-white appearance, with a bulk density ranging from 0.4 to 0.6 g/cm^3^ and a moisture content of ≤6.0%. The particle size of the powder varies between 1 and 50 µm, while dynamic light scattering (DLS) analysis indicates an average particle size of 150 nm. The CNC exhibits a conductivity of less than 350 µS/cm and a pH range of 5.0 to 8.0. Additionally, the viscosity of the CNC is measured at more than 5 cP. A bio-based polyol was synthesized by blending castor oil and glycerol in a weight ratio of 3:1. Isotane DM, a polymeric methylene diphenyl diisocyanate (p-MDI), was used as the NCO source. Additional chemicals included polyethylene glycol (PEG-400) as a chain extender, silicon oil as a surfactant, and dimethylbenzylamine as the catalyst.

The rigid polyurethane foams (RPUFs) were manufactured by mechanically mixing castor oil (24 parts per gram), distilled water, glycerol (8 parts per gram), PEG-400 (3.5 parts per gram), silicon oil (1 part per gram), and filler at 1000 rpm for 120 s. Afterward, p-MDI (63 parts per gram) and amine (0.4 parts per gram) were added to the mixture and stirred for an additional 60 s, maintaining an NCO/OH stoichiometric ratio of 1.2.

**Figure 1:**
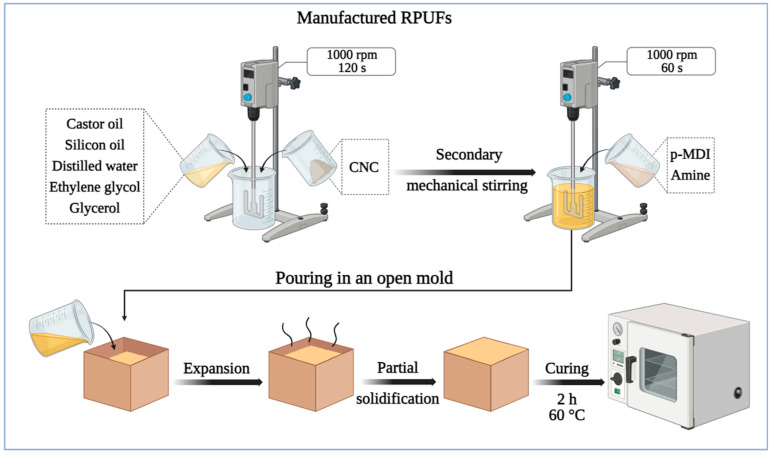
Flowchart of the foam manufacturing process.

The detailed formulation used for the RPUFs is presented in Table 1, following methodologies described in previous studies [16,19]. The reaction mixture was then cast into an open mold, allowing free expansion (Figure 1). The RPUF was cured at 60 °C for 2 h and post-cured at room temperature for two weeks. Filler weight fractions of 1%, 3%, and 5% were tested. The CNC contents selected in this study (1%, 3%, and 5% by weight) were defined based on previous research, which has reported effective reinforcement of PU foams using CNC concentrations typically ranging from 0.2% to 8% depending on the foam type and formulation conditions [8,14,15,20].

Morphological analysis of the RPUFs was performed using scanning electron microscopy (SEM) with MA10 equipment (Zeiss Evo, Oberkochen, Germany), operating at 3 kV, to observe the cell structure perpendicular to the rise direction. For each formulation, 30 cells were measured using ImageJ software (version 1.54g) to determine the average cell length (l) and width (w). The anisotropy index of the RPUFs was calculated following the method of Acosta et al. [21], where l and w are considered, based on the number of cells measured.

Compression testing of the RPUFs was performed parallel to the rise direction using seven prismatic samples per group (5.0 × 5.0 × 2.5 cm^3^). These tests were conducted using a 23-5D universal testing machine (Emic, Tokyo, Japan) with a crosshead speed of 2.5 mm/min, and maximum compressive strength was determined at 3.3 mm displacement, according to ASTM D1622 [22]. Apparent density was measured using the same samples with an analytical scale (0.001 g resolution) and a digital caliper (0.01 mm resolution). Storage modulus (E′) and tan delta (δ) curves were obtained by dynamic mechanical analysis (DMA) using a 2980 equipment (TA instruments, New Castle, DE, USA) operating in compression at 1 Hz frequency, 10% strain, with preload of 1 N and heating from −50 to 200 °C, at 3 °C·min^−1^. The tests were conducted using cylindrical samples (15 mm in diameter, 10 mm in height) under an inert N_2_ atmosphere.

### 2.2. Manufacturing and Characterization of Sandwich Panels

RPUFs containing 3% CNC were selected for the sandwich panels, as this percentage showed the best mechanical performance in prior tests. To obtain the confined RPUF condition, foaming was carried out in a prismatic wooden mold with internal dimensions of 30 × 30 cm and 10 cm in height. A mixture volume equivalent to 200% of the mold’s internal volume was poured, and the mold was then closed immediately to restrict foam expansion. This procedure resulted in a confinement ratio of 50%, meaning the foam was forced to expand under constrained conditions, thereby increasing its density and promoting a more compact cellular structure. This confinement method was performed as described by Kerche et al. [16]. Pine wood face sheets, 0.78 to 1 mm thick, were bonded to the foam cores using 20 g of commercial epoxy resin per side to adhere the face sheets to the core. These wood veneers, made from *Pinus elliottii* wood, were sourced from EcoFolhas (São Paulo, Brazil) and had an apparent density of 0.59 ± 0.05 g/cm^3^, a moisture content of 9.97 ± 1.99%, and a porosity of 49.25 ± 2.25%. Figure 1 illustrates the step-by-step manufacturing process for both the RPUFs and sandwich panels, highlighting the critical stages such as the mixing, molding, curing, and bonding of materials.

Flexural testing was carried out on prismatic samples (200 × 75 mm^2^) with a constant speed of 1 mm/min, in accordance with ASTM C393 [23]. The tests were performed using an Instron 3382 universal testing machine (Norwood, MA, USA), which ensured accurate load application and data acquisition. A total of seven specimens were tested per group to ensure statistical relevance. Additional details regarding the testing configurations and the calculation of mechanical properties can be found in the literature [19,24].

### 2.3. Statistical Analysis

All data were analyzed using ANOVA tests. When the null hypothesis was rejected, Tukey’s tests were applied to compare the means. Prior to this, the homogeneity of variances and normality of data were verified using Levene’s and Shapiro–Wilk tests, respectively. Statistical significance was determined at a 5% level.

## 3. Results and Discussion

**Figure 2:**
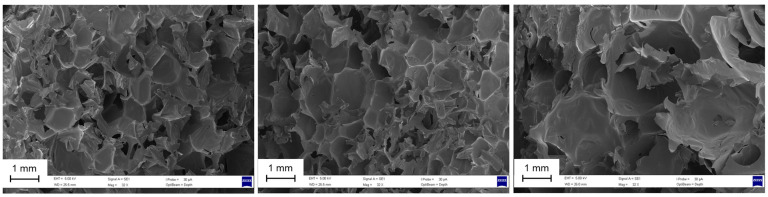
SEM images of RPUFs containing 1%, 3%, and 5% CNC.

Figure 2 presents the SEM images of RPUFs containing 1%, 3%, and 5% CNC. Besides, Table 1 summarizes the morphological properties, including cell dimensions and anisotropy index, for the different CNC contents. The micrographs reveal a predominantly closed-cell morphology across all formulations, which is a desirable feature for thermal insulation and structural applications of rigid foams. In the RPUF with 1% CNC, the cell structure appears more compact, with smaller and relatively uniform cells. This indicates that the addition of a low CNC concentration does not disrupt the foaming process or cell formation. As the CNC content increases to 3%, the cells become slightly larger and show improved uniformity in shape and distribution. This can be attributed to CNCs acting as effective nucleating agents, facilitating more consistent cell growth [25]. At this concentration, the foam also exhibits more elongated cells, which is reflected in the increased anisotropy index.

In the RPUF with 5% CNC, the micrographs show a pronounced increase in cell size—up to 40% larger compared to the 1% sample—with more defined cell walls and occasional signs of irregularity. While the overall structure remains closed-cell, the increased CNC loading appears to lead to slight cell wall thinning in some regions, possibly due to particle agglomeration. No free CNC particles were observed, indicating satisfactory interaction with the polymer matrix; however, the internal distribution may not be fully homogeneous at higher concentrations. These observations align with previous studies by Acosta et al. [21], who reported improved cell distribution and size regulation when using cellulose-based fillers, and Coccia et al. [26], who demonstrated that well-dispersed CNC enhances foam homogeneity.

It is worth noting that previous studies using the same RPUF formulation adopted in the present work have reported morphological data for unfilled control foams that provide a useful baseline for comparison. Acosta et al. [21] reported average values of 569 µm for cell length, 436 µm for cell width, and an anisotropy index of 1.34, while in a subsequent study, Acosta et al. [27] found slightly higher values of 630 µm, 615 µm, and 1.01, respectively, for the same unreinforced polyurethane system. When compared to the CNC-reinforced foams in the present study, which exhibited cell dimensions up to 1.54 mm in length and 1.10 mm in width, but anisotropy index values below 0.13, a clear pattern emerges: CNC incorporation promoted substantial cell expansion while simultaneously enhancing morphological symmetry. Both cell length and width increased with CNC content, which correlates with the higher degree of cell expansion observed in the SEM images. For instance, the cell length increased by 78% from the 1% to the 5% CNC sample. Statistical analysis indicated a significant increase in cell length for the 5% CNC sample, while the anisotropy index increased by 35% from 1% to 3% and again by 15% from 3% to 5%. This increase in the anisotropy index can be explained by the role of CNCs in promoting preferential nucleation along specific directions during cell formation, resulting in more elongated and irregular cell structures. This observation aligns with previous studies, such as those by Septevani et al. [14], which suggest that the reinforcement with particles can alter the balance between cell expansion and the orientation of cells within the polymer matrix.

Similar morphological trends have been reported in recent studies employing CNCs in polyurethane foam systems. Ugarte et al. [15] investigated flexible PU foams synthesized from castor oil-based polyols reinforced with varying CNC contents (0.5–1.5 wt%). They observed a progressive increase in average cell radius, from approximately 305 µm in the control sample to 389 µm in the foam with 1.5% CNC. SEM analysis revealed more voluminous cells and thinner walls, while advanced techniques such as scattering-type Scanning Near-Field Optical Microscopy (s-SNOM) and Fourier Transform Infrared Nanospectroscopy (nano-FTIR) confirmed that CNCs were embedded within the cell walls and contributed to structural reinforcement. Septevani et al. [14] studied RPUFs containing 0.2–0.8 wt% CNCs and found that 0.4% was the optimal concentration for morphological refinement. At this level, CNCs acted as efficient nucleating agents, promoting smaller, more uniformly distributed closed cells. Their study highlighted the importance of good interfacial compatibility and homogenous CNC dispersion, which were achieved through a solvent-free ultrasonication method. In contrast, Huang et al. [9] developed high bio-content RPUFs using 40% bio-polyol from microwave liquefaction of rape straw and reinforced them with 1–6 wt% CNCs extracted from liquefaction residues. At 4% CNC, SEM images showed improved regularity and thicker cell walls; however, higher concentrations led to irregular morphology and signs of structural disruption, attributed to excessive cross-linking and CNC agglomeration. These findings from the literature confirm that CNCs influence foam morphology in a highly concentration-dependent manner and that optimal effects—such as improved cell uniformity and wall integrity—occur only within a narrow compositional window.

**Figure 3:**
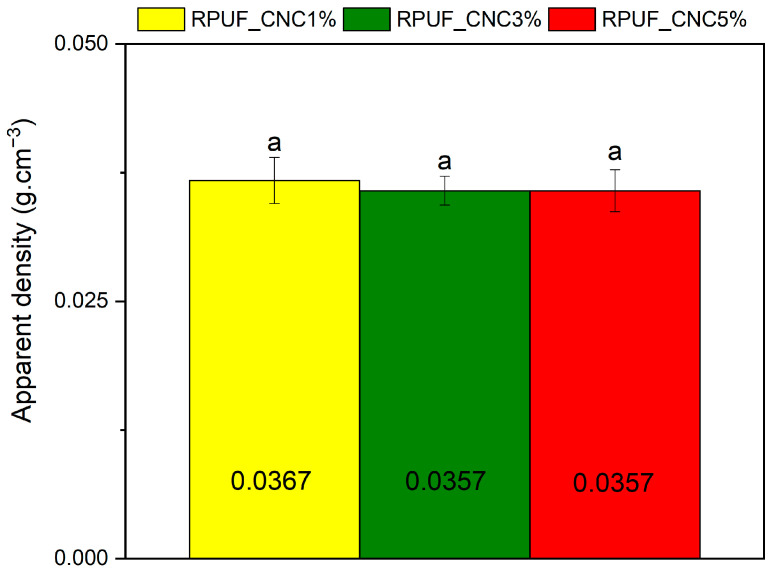
Apparent density of RPUFs containing 1%, 3%, and 5% CNC. Where: same letters above the bars mean significantly equal averages.

Figure 3 shows that the apparent density of the RPUFs remained relatively stable across the different CNC contents. The apparent density varied by less than 5% across the different CNC contents. Notably, Acosta et al. [21] and Acosta et al. [27] reported an apparent density of approximately 0.04 g/cm^3^ for neat RPUF (without any filler), a value that is very close to those obtained in the present study, further supporting the assertion that CNC addition did not compromise foam expansion or volumetric stability. This consistency suggests that CNCs while affecting cell structure, did not significantly alter the overall density of the foam. One plausible explanation is that CNCs contributed to a more uniform cellular structure without introducing substantial changes in mass distribution. Similar results were found in studies by Septavani et al. [14], where CNC-reinforced RPUFs maintained a stable density despite variations in CNC content. This result implies that the mechanical reinforcement achieved through CNC addition does not come at the cost of increased weight, which is an essential consideration in applications where low weight and high strength are critical, such as in insulation panels and lightweight structural components.

The stability of apparent density observed in this study is consistent with findings reported in several previous works investigating the incorporation of CNCs into polyurethane foams. In semi-rigid foams based on palm oil polyol, Zhou et al. [8] found that the addition of CNCs in concentrations ranging from 1% to 8% did not lead to significant changes in density, which was attributed to the good compatibility of the matrix and the balanced kinetics of expansion and gelation. Similarly, Coccia et al. [26] reported that flexible polyurethane foams containing 0.5% and 1% of surface-functionalized CNCs exhibited densities nearly identical to the unmodified foams. This was interpreted as an indication that the chemical crosslinking introduced by the CNCs did not significantly alter the overall foaming expansion. In a more recent study, Fontana et al. [20] compared two different CNC functionalization routes (freeze-drying vs. solvent exchange) for bio-based rigid PU foams and found that, regardless of the method or CNC-polyol interactions, the density of the foams containing 5 wt% CNCs remained close to that of the neat formulation. This was true even in cases where dispersion was less effective, suggesting that well-integrated CNCs do not compromise foam expansion volume.

**Figure 4:**
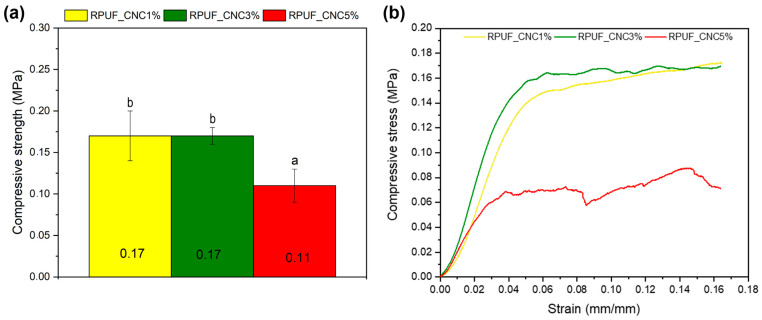
Compressive strength (**a**) and representative stress-strain curves (**b**) of RPUFs containing 1%, 3%, and 5% CNC. Where: Different letters above the bars mean statistically different averages.

Figure 4 depicts the compressive strength of the RPUFs at different CNC contents. Interestingly, the compressive strength for the RPUFs reinforced with 1% and 3% CNC was quite similar, while the strength of the RPUF with 5% CNC was lower. Compressive strength decreased by approximately about 55% in the 5% CNC sample compared to the 1% CNC sample. This trend could be attributed to the agglomeration of CNCs at higher concentrations, which may have led to weak points within the foam structure, as noted in the work of Septevani et al. [14]. At lower CNC concentrations (1% and 3%), the nanoparticles likely dispersed more uniformly, reinforcing the cell walls effectively. However, at 5%, CNC agglomerates may have disrupted the foam’s uniformity, leading to reduced mechanical performance. Similar phenomena have been observed in nanocomposites where excessive nanoparticle content hinders rather than enhances mechanical properties [8]. It is important to note that the control formulation used in the present study is the same control foam previously reported by Acosta et al. [21] and Acosta et al. [27], who manufactured using the same procedure and composition. In that earlier work, the compressive strength of the control foam was reported to be approximately 125–130 kPa, which is slightly higher than the values obtained in the current work. This minor difference is not due to changes in formulation or processing, but rather to natural variability between sample batches and test conditions. Therefore, the results remain consistent and further validate the mechanical behavior trends observed here.

The representative stress-strain curves presented in Figure 4b reveal the typical behavior of RPUFs under compressive loading. In all formulations, the curves show an initial relatively linear elastic region up to approximately 0.04 mm/mm of strain, followed by a plateau region that extends up to around 0.17 mm/mm, at which point the test was interrupted. This plateau indicates the gradual collapse of the cellular structure, an aforementioned phenomenon. The similarity in the elastic region across samples suggests that CNC incorporation at 1% and 3% had minimal impact on initial stiffness. However, the curve for the 5% CNC formulation presents a lower stress level throughout the compression range, confirming the mechanical weakening associated with excessive CNC content, likely due to reduced cell wall integrity or local inhomogeneities.

The nonlinear behavior observed in the initial portion of the stress-strain curves (Figure 4) is attributed to the progressive collapse of the foam’s cellular structure. This phenomenon, common in rigid polyurethane foams, results from localized buckling and fracture of the cell walls under compressive loading [28]. Once deformation initiates, the stress is redistributed through adjacent cells, leading to a gradual but irreversible loss of structural integrity. Accordingly, this collapse is considered destructive, as the foam does not recover its original shape after unloading. This interpretation is consistent with previous studies on rigid cellular polymers, where compression beyond the elastic regime leads to permanent microstructural damage rather than recoverable viscoelastic deformation [28,29]. For this reason, a second compression test on the same sample would not yield meaningful results and was therefore not performed.

A similar non-linear trend in compressive performance due to CNC content has been reported by other researchers. Zhou et al. [8] investigated semi-rigid biopolyurethane foams reinforced with CNCs at concentrations of 1%, 2%, 4%, and 8% (phr). They reported that compressive strength increased from 54 kPa (neat foam) to 117 kPa at 4% CNC, but then plateaued or slightly declined at 8% CNC due to densification and possible CNC clustering. In the study by Fontana et al. [20], rigid bio-based polyurethane foams containing 5 wt% of functionalized CNCs exhibited compressive strength values ranging from 160 to 220 kPa, depending on the method of CNC functionalization. The best performance was obtained with CNCs modified via freeze-drying and solubilization in DMA/LiCl, which ensured better dispersion. Conversely, when CNCs were prepared via solvent exchange and showed poor dispersion, the compressive strength dropped despite identical filler content. Huang et al. [9] observed that in RPUFs with 40% bio-based polyol, the compressive strength increased with CNC addition up to 4%, reaching approximately 150 kPa, but then decreased at 6% CNC, with values falling below 130 kPa. The drop was attributed to foam structural heterogeneity and localized stress concentrations introduced by CNC agglomerates. These findings support the interpretation that CNCs contribute effectively to foam reinforcement only within an optimal concentration window—typically below 4–5%—beyond which their agglomeration and poor matrix integration compromise mechanical performance, as was observed in the present study with the 5% CNC formulation.

**Figure 5:**
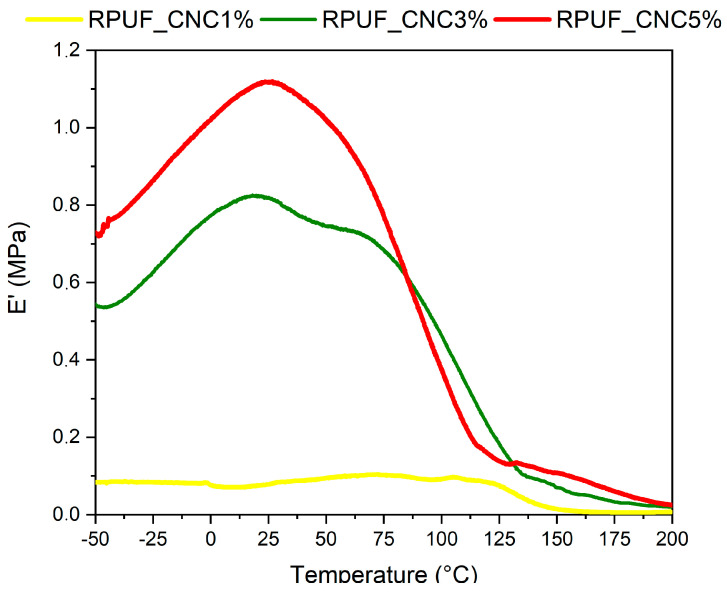
Storage modulus (E’) vs. temperature curves for RPUFs containing 1%, 3%, and 5% CNC.

Figure 5 displays the storage modulus (E′) versus temperature for the PU foams with different CNC contents. As the CNC content increased, the storage modulus rose, indicating improved stiffness across a broad temperature range. All samples showed pronounced peaks between −50 °C and 125 °C, suggesting that the incorporation of CNCs enhanced the foam’s rigidity, particularly at higher temperatures. Theoretical explanations for this behavior can be attributed to the stiffening effect of CNCs, which have a high modulus and contribute to load transfer within the foam matrix. Additionally, as CNCs are thermally stable, they likely improve the foam’s resistance to thermal softening, as demonstrated by previous research from Coccia et al. [26]. The sharp increase in modulus with CNC content highlights the potential of CNC-reinforced foams for high-temperature applications, such as in automotive and construction materials, where thermal stability and mechanical integrity are paramount.

**Figure 6:**
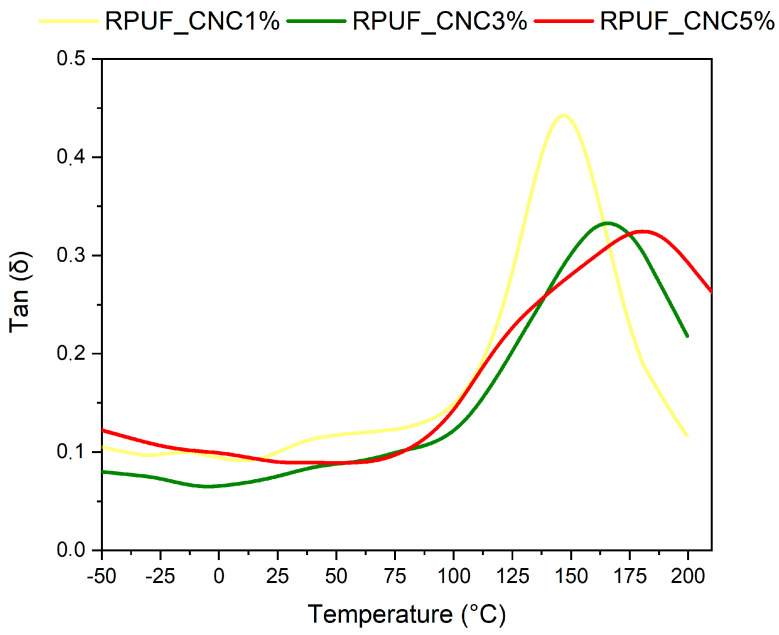
Tan Delta vs. temperature curves for RPUFs containing 1%, 3%, and 5% CNC.

Figure 6 shows the tan delta curves, indicating the damping properties of the RPUFs. That RPUF with 1% CNC exhibited a sharper peak, with a maximum of around 150 °C, while the other samples showed broader, less intense peaks with maxima near 170 °C. This shift suggests that the incorporation of higher CNC content results in a more rigid structure with reduced damping capacity, as the CNCs limit the molecular mobility of the polyurethane chains. These findings are in line with research by Zhou at al. [30], who demonstrated that higher nanoparticle content in polymer matrices tends to restrict chain movement. The lower tan delta values at higher CNC contents indicate that the RPUFs with 3% and 5% CNC would be less prone to energy dissipation, which is advantageous for applications requiring materials with high energy storage and low loss, such as vibration-damping components.

The dynamic mechanical results observed in the present study are in good agreement with the findings of Ugarte et al. [15], who performed a detailed DMA analysis on flexible polyurethane foams reinforced with CNCs. In that study, the addition of CNCs (0.5%, 0.75%, and 1.5%) led to a notable increase in storage modulus (E′) across the entire temperature range (−100 °C to 200 °C). For example, at 25 °C, E′ increased significantly for PF100-0.5 compared to the neat PF100 formulation, reflecting a stiffening effect directly linked to CNC incorporation. Additionally, tan δ curves revealed a consistent trend: the peak height decreased and the peak broadened as CNC content increased, indicating reduced molecular mobility and a more constrained polymer network. This shift suggests a higher degree of interaction between CNCs and the polyurethane matrix, which limits energy dissipation and promotes energy storage—a behavior analogous to that observed in our samples containing 3% and 5% CNC. Ugarte et al. [15] also noted a decrease in thermomechanical stability at the highest CNC content (1.5%), likely caused by nanoparticle agglomeration, which is consistent with the drop in performance seen in our 5% CNC formulation. These parallels reinforce that the optimal thermomechanical enhancement from CNCs occurs at moderate concentrations, beyond which dispersion challenges may compromise the reinforcing effect.

**Figure 7:**
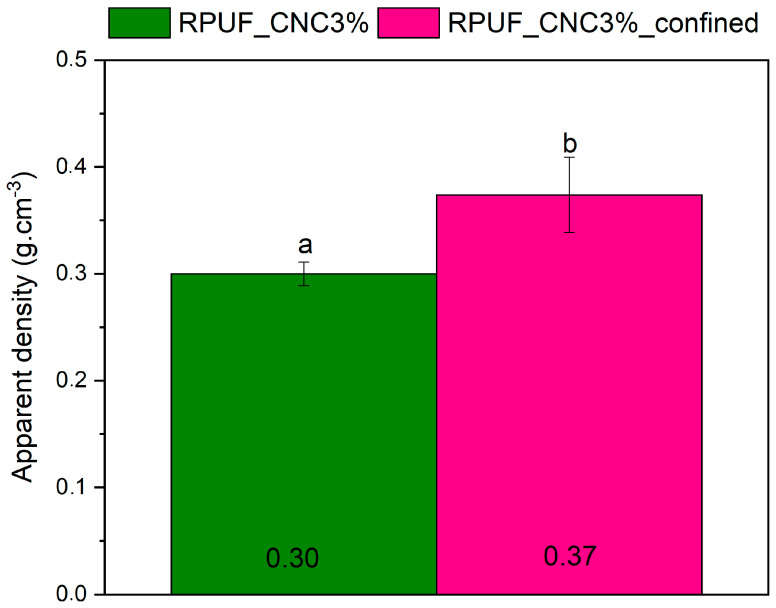
Apparent density of confined and unconfined sandwich panels with 3% CNC. Where: Different letters above the bars mean statistically different averages.

Based on the aforementioned results, the 3% CNC content was selected for the manufacturing of the sandwich panels with and without confinement because it demonstrated the best balance between mechanical strength and uniform cellular structure, without the agglomeration issues observed at higher CNC concentrations. This allows for a clear evaluation of the confinement effect on the foam’s properties, isolating the impact of confinement from the reinforcement benefits provided by CNC. Figure 7 compares the apparent density of the confined and unconfined sandwich panels. The confined panels exhibited a higher density (23%) due to the restricted expansion during the manufacturing process, which compressed the foam structure and increased the material’s packing density. This result is consistent with the findings of Kerche et al. [16], where confined polyurethane foams showed superior mechanical properties due to increased density. In practical terms, the higher density of confined panels translates to better load-bearing capabilities, making these panels more suitable for structural applications such as flooring or roofing where both strength and lightweight properties are needed.

**Figure 8:**
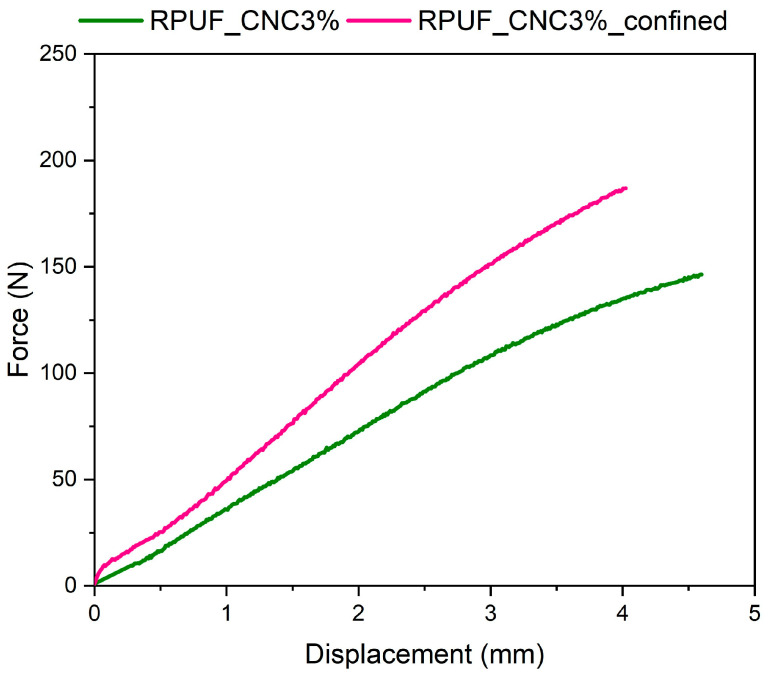
Force vs. displacement curves in flexural testing of confined and unconfined sandwich panels with 3% CNC.

The flexural response of the sandwich panels is presented in Figure 8. In the panels evaluated in this study, a non-linear behavior was observed in the elastic region of the stress-strain curve, which is typical in rigid foams. This non-linearity arises due to the cellular structure of the foam, where deformation mechanisms are more complex compared to solid materials, as described in the literature [19]. Therefore, as stress is applied, the foam’s cell walls begin to bend and buckle, gradually changing the stiffness. Unlike solid materials that show a linear elastic response, the bending and collapsing of foam cells cause a progressive increase in stiffness, leading to a non-linear stress-strain relationship even before the onset of plastic deformation. This characteristic behavior is governed by the internal structural collapse of the foam rather than simple elastic stretching. The confined panels demonstrated higher force-displacement values, indicating superior performance under flexural loading. This is likely due to the denser core and improved adhesion between the face sheets and core material, as shown in previous studies by Nar et al. [31], where confined RPUFs exhibited improved flexural properties. The higher flexural strength in confined panels suggests they are better suited for applications requiring strength under bending, such as wall panels and structural supports in lightweight buildings.

**Figure 9:**
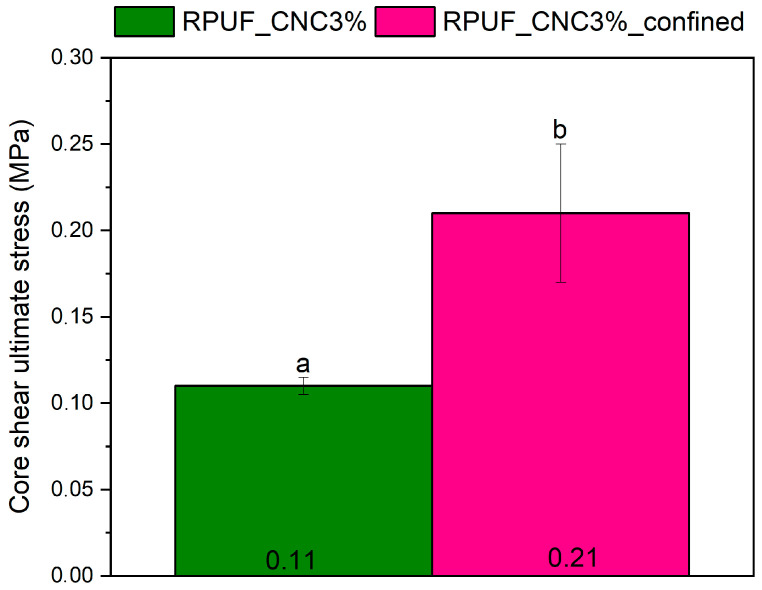
Core shear ultimate stress of confined and unconfined sandwich panels with 3% CNC. Where: Different letters above the bars mean statistically different averages.

**Figure 10:**
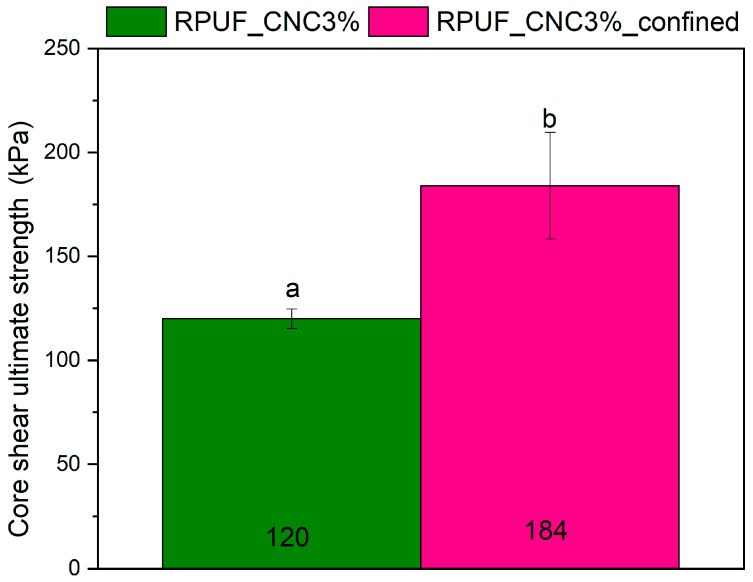
Core shear ultimate strength of confined and unconfined sandwich panels with 3% CNC. Where: Different letters above the bars mean statistically different averages.

Figure 9 illustrates the core shear ultimate stress of both confined and unconfined panels. The confined panels displayed significantly higher stress values (about 90%), likely due to the increased density and stronger interface between the core and face sheets. On the other hand, Figure 10 presents the ultimate shear strength of the core material in both confined and unconfined configurations. Confined panels exhibited higher strength (around 55%), which can be attributed to the reduced porosity and increased interaction between the core and the face sheets. Analogy results were obtained by Septevani et al. [14] and Zhou et al. [8] in CNC-reinforced RPUFs, where increased nanoparticle content enhanced the compression strength of the material. These findings indicate that confined panels can be used in environments where high shear loads are expected, such as in load-bearing wall panels or bridge decks, where resistance to core shear failure is critical.

**Figure 11:**
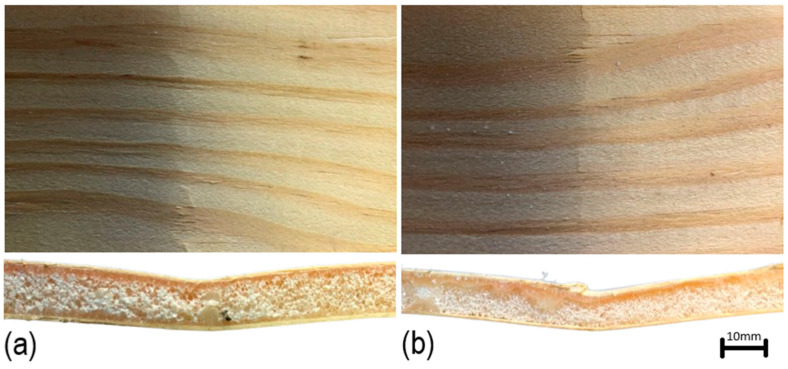
Top and lateral views showing failure modes of confined (**a**) and unconfined (**b**) sandwich panels with 3% CNC.

Figure 11 displays the failure modes of confined and unconfined sandwich panels during flexural testing. As expected, it is possible to observe a clear confirmation of the reduction in cell size due to confinement, as evidenced by the more compact cellular structure in the confined panels compared to the unconfined ones. All panels failed by crushing at the compression face, which is a typical failure mode in sandwich structures under bending loads. Theoretically, in flexural tests, the top face of the sandwich panel experiences compressive stress, while the bottom face is under tension. When the compressive stress in the top face exceeds the material’s compressive strength, localized crushing occurs. This failure mode, as observed, is consistent with the work of Delucis et al. [19], where similar flexural behaviors were reported in sandwich panels with rigid foam cores. From a practical perspective, this result indicates that the performance of the sandwich panels under bending is highly dependent on the strength of the face sheets and the core’s ability to resist compressive forces. In confined panels, the additional core stiffness may help distribute the compressive loads more effectively, delaying the crushing failure. This has significant implications for applications, such as in flooring systems, roofing panels, or other structural elements where the sandwich construction is used to maximize strength while minimizing weight.

The mechanical improvements observed in the confined sandwich panels—such as the increase in flexural strength and core shear performance—can be attributed to structural changes induced by the confined expansion process. This association between confinement and enhanced mechanical behavior is supported by morphological data reported in previous studies. For instance, Kerche et al. [11] demonstrated that applying a 50% confinement during foaming led to a significant reduction in average cell size, a more homogeneous distribution of cells, and thicker cell walls in both neat and fiber-reinforced RPUFs. These microstructural changes were directly correlated with improvements in compressive strength and modulus. Moreover, in CNC- or MFC-reinforced formulations, confined foams displayed more isotropic cellular structures, with lower anisotropy index values and fewer irregularities compared to unconstrained counterparts [16]. Such morphological refinement helps explain the enhanced mechanical response seen in the present study, confirming that confinement modifies the internal architecture of the foam in ways that are mechanically advantageous.

## 4. Conclusions

This study demonstrated that both CNC reinforcement and expansion under confinement play critical roles in enhancing the mechanical performance and structural integrity of rigid polyurethane foams (RPUFs) used in sandwich panels. The 3% CNC content proved to be the optimal concentration, offering a significant improvement in cellular structure and mechanical properties without the detrimental effects observed at higher CNC concentrations. Specifically, the 5% CNC content, while leading to a 78% increase in cell length, resulted in a 55% reduction in compressive strength. This highlights the importance of carefully balancing the CNC content to achieve the desired mechanical properties without compromising foam uniformity.

In addition to CNC reinforcement, confining the RPUF during its expansion resulted in a notable increase in RPUF density by 23%, which translated into significant gains in both core shear stress (90%) and core shear strength (55%) compared to unconfined RPUF s. The improvement in mechanical performance is attributed to the denser cellular structure and enhanced interface between the core and face sheets in the confined panels. These advancements suggest that combining CNC reinforcement with foam confinement can significantly enhance the load-bearing capacity and durability of sandwich panels, making them suitable for demanding structural applications, such as flooring, roofing, and walls in lightweight construction systems. Future studies may address additional parameters, particularly the closed-cell content and thermal conductivity, which are critical for insulation applications.

## Figures and Tables

**Table 1:** Morphological properties of RPUFs based on SEM Images.

Group	L (mm)	w (mm)	R
RPUF_CNC1%	0.870 (0.165 ^a^)	0.696 (0.213 ^a^)	0.096 (0.010 ^a^)
RPUF_CNC3%	1.057 (0.263 ^a^)	0.779 (0.215 ^ab^)	0.113 (0.039 ^b^)
RPUF_CNC5%	1.543 (0.619 ^b^)	1.097 (0.745 ^b^)	0.130 (0.056 ^c^)

Where: L is cell length; w is cell width and R is anisotropy index (in the same column, different letters next to the standard deviation represent significant differences).

## Data Availability

The original contributions presented in this study are included in the article. Further inquiries can be directed to the corresponding author.
